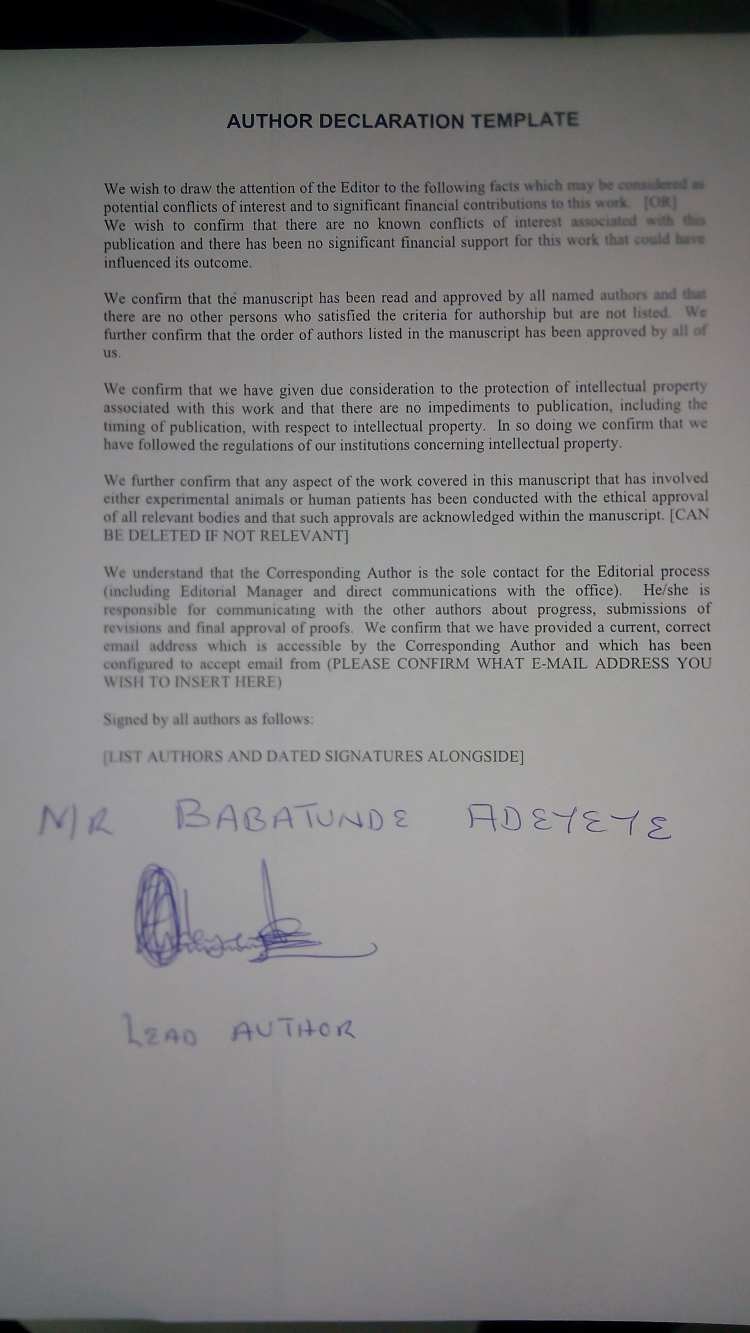# Data on new media use for agricultural training and research at Agricultural Services and Training Centre (ASTC)

**DOI:** 10.1016/j.dib.2018.11.031

**Published:** 2018-12-03

**Authors:** Babatunde Adeyeye, Lanre Amodu, Oscar Odiboh, Nelson Okorie, Evaristus Adesina, Darlynton Yartey, Thelma Ekanem

**Affiliations:** Department of Mass Communication, Covenant University, Nigeria

**Keywords:** Agriculture, Extension workers, New media, Research, Training

## Abstract

Data available in this survey depict new media use for agricultural training and research. The objective was to determine how favourably or unfavourably disposed the respondents were to using new media for research and training. Self-administered copies of the questionnaire were used to generate the data from 47 agricultural extension workers at ASTC, Plateau State. Frequency tables and the Spearman’s rank-order correlation were used to analyze data.

**Specifications table**

**Table:** 

Subject area	*Development Communication*
Specific subject area	*Agricultural Communication*
Type of data	*Primary data (Tables)*
How data was acquired	*Survey carried out with the use of a questionnaire*
Data format	*Raw, analyzed (SPSS)*
Experimental factors	*Data for this survey was gathered from forty seven respondents that were agricultural extension workers at ASTC. The questionnaire, being the instrument for the survey, was designed specifically for the research. Tables and the Spearman’s rank-order correlation were used to analyze the data.*
Experimental features	*The data provide information on respondents’ use of new media for research and training of farmers at the centre.*
Data source location	*Plateau State, North-Central Nigeria*
Data accessibility	*Dataset is included*

**Value of the data**

• Data can provide information on how agricultural extension workers use new media in training farmers.
• The data can provide insight into the relationship that exists between extension workers use of new media for farmers’ training and the rate at which the farmers adopt innovations at ASTC.
• The analyzed data can reveal whether or not there is a correlation between how extension workers source for training materials online and how they train the farmers.

## 1 Data

This data set provides information to key dimensions to how new media have been used for agricultural training and research at Agricultural Services and Training Centre (ASTC). New media provides diverse options for agricultural communicators as well as researchers and have become an important sphere of the experiences of modern societies. With new media, access to agricultural information relating to farmers training and research can be easily gotten online.

Data set supply information using tables on respondents’ use of the internet to source materials to train farmers. Correlation also provided information into the relationship between the frequency of respondents use of internet to source materials to train farmers and the general rate of adoption of innovations by farmers using new media; as well as the relationship between respondents use of internet to source materials to train farmers and how new media have been used to train farmers.

The dataset focuses on respondents’ use of new media for agricultural training and research. After a survey was conducted to obtain the responses of the participants, a percentage table was used to show respondents’ use of internet to source materials to train farmers. Correlation was also used to test the relationship between the frequency of respondents use of internet to source materials to train farmers and the general rate of adoption of innovations by farmers using new media; as well as the relationship between respondents use of internet to source materials to train farmers and how new media have been used to train farmers. Table 1 presents the data of extension workers use of internet to source materials to train farmers while Tables 2 and 3 present the data of the correlation tests.

**Table 1:** Respondents’ use of internet to source materials to train farmers.

	**Percent (%)**
**Yes**	91.5
**No**	8.5
**Total**	100%
**n**	47

**Table 2:** Correlation test between internet use and general rate of adoption of agricultural innovations.

			Respondents use of internet to source materials to train farmers	General rate of adoption of innovations by farmers using new media
Spearman׳s rho	Respondents use of internet to source materials to train farmers	Correlation Coefficient	1.000	−.180
		Sig. (2-tailed)	.	.225
		N	47	47
	General rate of adoption of innovations by farmers using new media	Correlation Coefficient	−.180	1.000
		Sig. (2-tailed)	.225	.
		N	47	47

**Table 3:** Correlation test between internet use and farmers training.

			Respondents use of internet to source materials to train farmers	How new media have been used to train farmers
Spearman׳s rho	Respondents use of internet to source materials to train farmers	Correlation Coefficient	1.000	−.087
		Sig. (2-tailed)	.	.559
		N	47	47
	How new media have been used to train farmers	Correlation Coefficient	−.087	1.000
		Sig. (2-tailed)	.559	.
		N	47	47

## 2 Experimental design, materials and methods

Survey was used to generate data from 47 respondents from Agricultural Services and Training Centre (ASTC), Jos, Plateau State Nigeria. The total number of extension workers and researchers at the centre was used; hence, census technique was adopted for the sampling. A self-designed questionnaire by the researchers was used to gather the data for the research and the Statistical Package for Social Science (SPSS) was used for the analysis.

### 2.1 Ethical considerations

Consent was gotten from the respondents to conduct the research and the participants were intimated on the intent of the study. The questionnaire was prepared in a way to guarantee the anonymity of the respondents as they were not required to offer any form of personal identification. Strict confidentiality of the responses was also given with the assurance that they data are entirely for research purposes.

## Figures and Tables

**Fig. S1:**