# Supplementary material for: Data on new media use for agricultural training and research at Agricultural Services and Training Centre (ASTC)
Source: Data Brief. 2018 Dec 3;22:181–4. doi: 10.1016/j.dib.2018.11.031 (PMC6302137; doi:10.1016/j.dib.2018.11.031)
Supplement: Supplementary file 1 — Supplementary material [file mmc2.docx]

Department of Mass Communication,

College of Business and Social Sciences,

Covenant University, Ota

Ogun State

Dear Respondent,

**REQUEST TO COMPLETE QUESTIONNAIRE**

We are researchers from the above department and university. We are carrying out a research on; **New Media Use for Agricultural Training and Research at Agricultural Services and Training Centre (ASTC)**

We solicit your co-operation in filling this questionnaire, while assuring you that all information provided shall be treated with confidence and used for academic purpose only.

Yours faithfully.

**QUESTIONNAIRE**

Please tick in the box that provides the most applicable option.

**1. Age**

Below 20 [ ]

21-30 [ ]

31-40 [ ]

41-50 [ ]

51 plus [ ]

**2. Gender**

Male [ ]

Female [ ]

**3. Marital Status**

Single [ ]

Married [ ]

Divorced [ ]

Widowed [ ]

**4. Educational Qualification**

Post Graduate [ ]

Degree/HND [ ]

ND/NCE [ ]

SSCE [ ]

First School Cert [ ]

None [ ]

**5. Do you think the use of internet and mobile phones affect the diffusion of agricultural innovations positively at ASTC?**

Yes, it does [ ]

No, it doesn’t [ ]

Not sure [ ]

**6. Do you use the internet in sourcing materials for training and research at ASTC?**

Yes [ ]

No [ ]

**7. How have you used the internet to train farmers at ASTC?**

To source for training information [ ]

Illustrations [ ]

Research [ ]

Form notes [ ]

Others, specify…………………

**8. What is the general rate of adoption of innovations by farmers trained at ASTC using new media options?**

High [ ]

Low [ ]

Zero [ ]
